# Nanocellulose-Based Conductive Membranes for Free-Standing Supercapacitors: A Review

**DOI:** 10.3390/membranes9060074

**Published:** 2019-06-25

**Authors:** Helen H. Hsu, Wen Zhong

**Affiliations:** Department of Biosystems Engineering, University of Manitoba, 75A Chancellor’s Circle, Winnipeg, MB R3T2N2, Canada; umxu68@myumanitoba.ca

**Keywords:** nanocellulose, energy storage devices, supercapacitors, membrane electrode

## Abstract

There is currently strong demand for the development of advanced energy storage devices with inexpensive, flexibility, lightweight, and eco-friendly materials. Cellulose is considered as a suitable material that has the potential to meet the requirements of the advanced energy storage devices. Specifically, nanocellulose has been shown to be an environmentally friendly material that has low density and high specific strength, Young’s modulus, and surface-to-volume ratio compared to synthetic materials. Furthermore, it can be isolated from a variety of plants through several simple and rapid methods. Cellulose-based conductive composite membranes can be assembled into supercapacitors to achieve free-standing, lightweight, and flexible energy storage devices. Therefore, they have attracted extensive research interest for the development of small-size wearable devices, implantable sensors, and smart skin. Various conductive materials can be loaded onto nanocellulose substrates to endow or enhance the electrochemical performance of supercapacitors by taking advantage of the high loading capacity of nanocellulose membranes for brittle conductive materials. Several factors can impact the electronic performance of a nanocellulose-based supercapacitor, such as the methods of loading conductive materials and the types of conductive materials, as will be discussed in this review.

## 1. Introduction 

Increasing environment concern has pushed the development of sustainable, eco-friendly, and biodegradable materials. Cellulose is considered as a renewable and multifunctional raw material that has the potential to replace many non-renewable materials [1,2]. With the development of nanotechnology, the isolation of nanocellulose from natural plants is becoming a heated topic in the field of advanced materials. The advantageous features of nanocellulose include its low density and high mechanical strength, Young’s modulus, and surface-to-volume ratio compared to synthetic materials [3]. The advanced features of nanocellulose membranes show great potential in many applications, such as wound dressing, membrane-based oil/water filters, and energy storage devices. Specifically, nanocellulose shows great potential to be used as substrates for loading conductive materials and fabricating lightweight flexible composite membrane electrodes with high electrochemical performance. These nanocellulose-based composite membrane electrodes can be assembled into energy storage devices with light weight, small size, flexibility, longer life cycles, and good electrical performance [4,5,6]. 

**Figure 1:**
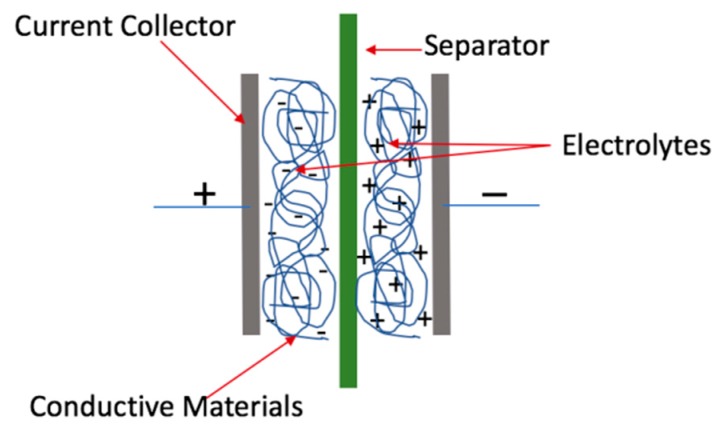
A typical electrical double-layer capacitor.

Supercapacitors (SCs) have attracted remarkable research interest in the past decade due to their higher energy storage capacity, higher power density delivery, and shorter charge–discharge time compared with conventional batteries (lead-acid or lithium-ion batteries) [5,6,7,8,9]. To meet the increasing demands on smart energy storage devices, conventional capacitors that are usually large, heavy, and inflexible are being replaced by lightweight micro-sized flexible SCs composed of flexible substrates such as nanocellulose membranes [7,10,11]. In general, there are two types of supercapacitors, including electrical double-layer capacitors (EDLC) and pseudocapacitors. Both types of SCs can be fabricated by nanocellulose membrane as the substrate. A typical EDLC consists of two metal plates (collectors), two electrodes membranes, one separator, and an electrolyte (Figure 1) [5,7,8,9]. Electrical energy is stored in EDLCs by separating positive and negative charges at the interface without faradaic reactions. A pseudocapacitor, on the other hand, has a reversible surface redox reaction on the surface of the electrodes’ materials. Pseudocapacitors generally have poor capability and low electrochemical conductivity as compared to EDLCs [7,10,11]. 

The electrochemical performance of an EDLC is mainly impacted by the total surface area of the electrode membranes for ion adsorption. A porous substrate usually allows significantly more conductive materials to be loaded on its surface as compared to a flat and solid substrate. Materials that have been used for porous substrates for electrode membranes include porous carbon materials, synthetic polymers, and nanocellulose. As compared to porous carbon materials, polymer substrates are usually more flexible, but they have a lower mass loading ratio than that of the conductive materials. Polyethylene and polypropylene are popular substrate materials to make electrode membranes; however, they are non-biodegradable and damage the environment. Therefore, extensive research has been devoted to develop biodegradable materials, including nanocellulose, to replace non-biodegradable materials for an EDLC electrodes membrane substrate. This review will start with a brief introduction of the existing technology of isolating nanocellulose from natural plants, and then focus on the development, evaluation, and applications of supercapacitors based on nanocellulose without thermal treatment (e.g., thermal pyrolysis or carbonization). Research and development on carbonized cellulose-based supercapacitors can be found in other recent studies [12,13,14,15] and reviews [16,17], but these areas are not within the scope of this review. This review will only cover conductive composite membranes with nanocelluose as a substrate for loading conductive agents.

## 2. Isolation of Nanocellulose from Natural Plants

### 2.1. Cellulose in Plants

**Figure 2:**
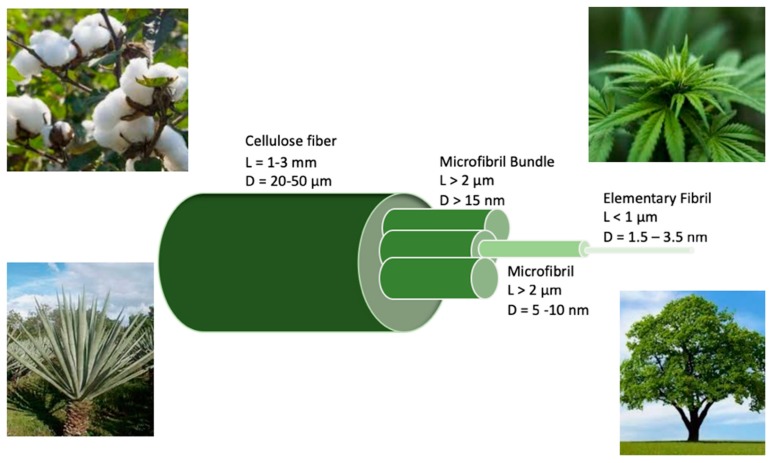
Hierarchical structures of cellulose fibers.

Woods, agriculture residues, and annual plants such as flax, hemp, sisal, rice, wheat, and pineapple are examples of many natural sources for cellulose [18] (Figure 2). Cellulose is the main component in the cell wall of plants in the form of microfibrils (5–10 nm), which are bundled to form cellulose fibers. Each single cellulose fiber is a component in the cellulose microfibrils; they are embedded in a matrix that are composed of lignin and hemicellulose [19]. Lignin cannot be hydrolyzed in acid; however, it can be immediately oxidized by alkaline under elevated temperature, and therefore can be easily removed with phenol [20]. Hemicellulose constitutes an amorphous structure that surrounds cellulose nanofibers and contains polysaccharides, which remain within the connections of cellulose after lignin is removed [20,21]. Hemicellulose is a different component from cellulose in that it has various units of sugars, a higher degree of branching, and a lower degree of polymerization than cellulose [20,21]. 

Nanofibrillated cellulose (NFC) and nanocrystals (CNC) are two major types of nanocellulose that have generally been obtained from different procedures [18]. NFC could be isolated from plants using such mechanical processes under high-pressure homogenization, grinding, and refining [22], while CNCs can been isolated by acid hydrolysis treatments [23]. NFC undergoes transverse cleavage in the amorphous regions under acid hydrolysis, and the use of sonication results in rod-like materials that are referred to as cellulose whiskers [24,25]. The properties of cellulose nanofibers derived from natural sources are influenced by several key factors such as chemical structures, internal fiber structures, microfibril angles, and cell dimensions, which differ among the different parts of a plant [26]. Natural cellulose fibers have higher mechanical strength than other natural fibers such as silk and wool, and have a Young’s modulus that is comparable to that of advanced fibers such as E-glass fibers (Table 1) [3,18]. 

### 2.2. Decortication

Decortication is an important process to remove impurities from plant fibers that has a critical impact on the quality of the produced fibers [27,28]. Traditional decortication methods start with separating the long bast fibers from the stems by dew or water retting; it takes around 20 days to degrade the pectin, hemicellulose, and lignin. Chemical and mechanical decortications are usually followed to treat retted materials. Chemical methods usually produce cleaner fibers, but cause concerns regarding environmental pollutions and safety issues during manufacturing [29,30,31]. Mechanical methods such as toothed breaking rollers have been used to decorticate bast fibers from raw plants such as hemp, flax, and linseed [27,28]. However, mechanical methods usually require dry and well-retted straws for the decortication process and may break the long fibers [27,28]. The advantage and disadvantages of various decorticate methods can be found in other reviews [29,30,31,32,33]. The developed bast fibers can be further processed to yield nanocellulose, as discussed in the following sections.

### 2.3. Mechanical Methods for Nanocellulose Isolation

Several mechanical methods are available to isolate nanocellulose from cleaned bast fibers or natural plants. Nanocellulose can be isolated from the secondary cell walls by the shearing method without causing degradation on the cellulose. The morphology and aspect ratio of the nanocellulose depend on the defibrillation techniques including refining, grinding, bleaching, ultrasonication, and homogenization [20]. Refining and high-pressure homogenizing processes are now performed by manufactures because of their high efficiency and productivity [34,35]. However, high energy consumption is a major drawback for mechanical processes. Cryocrushing is a method to obtain nanofibers by using liquid nitrogen to freeze the fibers, which is followed by high shear forces. It involves a combination of shearing processing in a refiner, and subsequent crushing in liquid nitrogen [36]. The fibers treated by this method are subsequently either freeze-dried or suspended in distilled water. 

### 2.4. Chemical Methods for Nanocelllulose Isolation

Chemical pre-treatments including alkaline, acid, oxidation, and the enzymatic treatment of cellulose are effective ways to decrease the energy consumption in the production of nanocellulose by mechanical methods. Alkaline treatments of bast fibers disrupt the lignin structure, and could help separate the linkages between lignin and carbohydrates [37]. Mild alkali treatments result in the solubilization of lignin, pectins, and hemicelluloses. Nanofibers were successfully extracted from jute fibers by alkali and dimethyl sulfoxide treatments followed by acid hydrolysis [38]. Nanofibers were also isolated from banana fibers by steam explosion at a temperature range from 220 °C to 300 °C, which causes the thermal depolymerization of hemicellulose and the cleavage of glycosidic linkages of cellulose [2]. Another commonly used pre-treatment is 2,2,6,6-tetramethyl-1-piperidinyloxy (TEMPO)-mediated oxidation, which introduces the functional groups: carboxylate and aldehyde in cellulose [39,40]. A comparative study was made between NFCs derived from eucalyptus and *Pinus radiata* pulp fibers via homogenization with or without TEMPO-mediated oxidation [41]. The NFC films with TEMPO-mediated oxidation showed less shrinkage and higher transparency levels than the other ones [41]. Enzymatic pre-treatment is another method to increase the yielding of nanocellulose [42]. For example, endoglucanase pre-treatment was found to facilitate the disintegration of wood fiber pulp into nanocellulose. Moreover, enzymatic pre-treated wood nanocellulose showed more a uniformed structure than nanocellulose produced by acid hydrolysis [34,43]. However, nanocellulose can be degraded after acidic hydrolysis, which therefore may affect the overall quality of the produced nanocellulose [44]. Electrospinning is a simple and rapid method to regenerate nanocellulose, and it can be potentially scaled up to the industrial level [45].

## 3. Nanocellulose-Based Supercapacitors

Most conductive agents such as polymers, metallic particles, and carbon are brittle, and are therefore usually used in combination with a soft and flexible substrate to fabricate lightweight and free-standing flexible membrane electrodes for supercapacitors. Nanocellulose with highly porous structures provides an ideal substrate for loading a large amount of conductive materials, and facilitates ions’ transit through their porous structures. Nanocellulose also has excellent mechanical performance with a tensile strength at 1.7 GPa and a Young’s modulus at 100–130 GPa, which are comparable to those of glass and aramid fibers [46]. In addition to supercapacitors, nanocellulose membranes have also been used as flexible substrates for other types of energy storage devices, including rechargeable lithium-ion batteries (LIBs) or solar cells [47,48]. These energy storage devices showed great potential on emerging power sources for wearable electronics, electric vehicles, or giant energy-storage systems. 

Recently, advanced technologies have been developed and applied on the designs of SCs to achieve “smart” functions including self-healable, foldable, shape memory, pH-sensitive, or thermosensitive [49,50,51,52]. Most of the electrodes membranes in smart SCs were fabricated by synthetic polymers or carbon-based materials to achieve superb mechanical strength and/or flexibility, so that they can be twisted, stretched, or bent into different shapes and can return to their original shape/size with or without stimuli [9]. 

**Figure 3:**
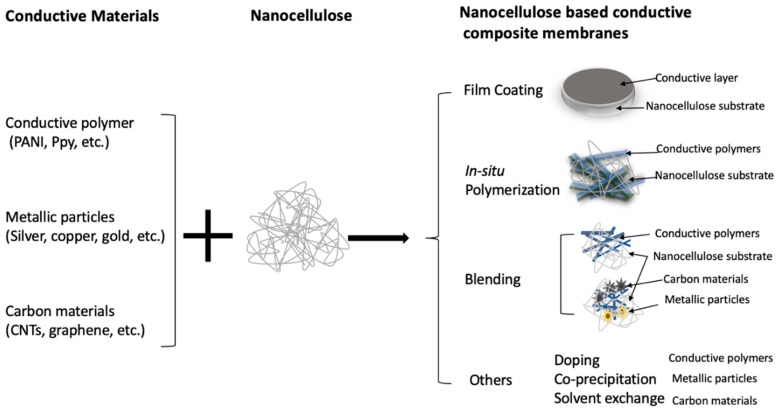
Fabrication routes of nanocellulose (NC)-based conductive composite membranes.

In order to enhance the electrochemical performance of the electrode membranes, a large amount of conductive materials is usually loaded onto the substrate, which may decrease the mechanical strength and flexibility of the membrane electrodes. It has been a challenge to keep an optimum balance between the electrochemical and mechanical performances of the composite membrane electrodes. Generally, there are two major procedures to incorporate conductive components into nanocellulose substrates to fabricate composite membrane electrodes: one is to coat conductive materials onto the top of nanocellulose matrix, and the other is to mix conductive agents into the nanocellulose substrate. Schematic illustrations of the fabrication process of nanocellulose-based conductive composite membranes are shown in Figure 3. The summary of nanocellulose-based conductive membranes developed for supercapacitors and energy storage devices are listed in Table 2. More details will be discussed in the following sections. 

### 3.1. Nanocellulose Supercapacitors Loaded with Metallic Particles

Metallic particles have a high intrinsic conductivity of about 10^5^ S/cm, which is five times higher than that of carbon-based conductive materials [53,54]. Both metallic particles and carbon materials are usually used for direct coating on the surface of nanocellulose substrate, which is a simple and convenient method for producing conductive nanocellulose-based composite membrane electrodes. However, the conductivity of the membrane electrodes is positively associated with the loading of the metallic materials or thickness of the coating layer [55]. However, metallic particles are brittle and heavy, and therefore are difficult to be dispersed evenly onto the substrate. One way to enhance the dispersion of the materials is to coat metallic particles onto the surface of the nanocellulose substrate. Various metal hydroxides/oxides grown on nickel foam were also coated onto nanocellulose films to fabricate flexible supercapacitor electrode materials with good conductive property [56]. Peng et al. (2017) developed flexible supercapacitors with high capacitance (385 F/g) by coating copper oxide and polypyrrole onto the bacterial cellulose substrates [48]. In most cases, coating conductive polymers or carbon materials onto nanocellulose membranes may decrease the transparency because the coated layer is not uniform on a large scale, leading to a significant light scattering. Hu et al. (2013) deposited a thin layer of tin-doped indium oxide, carbon nanotubes, and silver nanowires onto transparent nanocellulose substrates to fabricate transparent and conductive nanopapers with good conductivity (25 Ω sq^−1^) and power conversion efficiency (0.4%), which can be used for applications in optoelectronics devices such as displays, touch screens, and interactive papers [57].

### 3.2. Nanocellulose Supercapacitors Loaded with Conductive Polymers

Conductive polymers were introduced in 1977 [58], and found their applications in battery industries in 1980s as alternatives to metallic materials because of their good electrochemical performance, light weight, and low cost [59]. Polyacetylenes (PAC), polypyrroles (PPy), polyaniline (PANI), poly(p-phenylene vinylene) (PPV), poly(thiophene)s (PT), poly (3,4-ethylenedioxythiophene) (PEDOT), and poly (p-phenylene sulfide) (PPS) are widely used conductive polymers for fabricating electrodes of energy storage devices. Among the conductive polymers, PANI has been considered as the most promising conductive polymer for applications in SCs or battery electrode membranes, because of its simple route of synthesis, controllable conductivity, and high specific capacitance [60,61]. 

In situ polymerization and blending are the two major procedures to incorporate conductive polymers into nanocellulose membranes. The major advantages of in situ polymerization to incorporate conductive polymers into nanocellulose include simple fabrication procedures with low cost and uniform three-dimensional (3D) network structures, which contribute to the good electromechanical performance of the final composite membranes. Therefore, in situ polymerization has been an efficient method to grow PANI on a nanocellulose substrate to fabricate composite membranes. Specifically, nanocellulose is usually impregnated with the monomer in a solution, and then, a nanocomposite of nanocellulose/PANI can be produced by the addition of an initiator such as ammonium persulfate to intrigue the in situ polymerization. 

The in situ polymerization strategy is also suitable for other conductive polymers such as PPy and PPV. A recent study reported the fabrication of conductive nanocellulose composite films composed of poly (3,4-ethylenedioxythiophene) via in situ polymerization: polystyrene sulfonate (PEDOT:PSS) and polypyrrole (PPy). Its electrical conductivity reached 10.55 S/cm, and its specific capacitance reached 315.5 F/g [62]. Moreover, nanocellulose-based PEDOT:PSS-PPy nanopapers showed higher flexibility than the nanopapers containing only polypyrrole [62]. Abundant studies have been conducted to develop electrodes using conductive polymers such as PANI, PPy, and PPV along with nanocellulose to assemble composites that have both good electrochemical and mechanical properties [48,62].

An acidic environment is usually required during the in situ polymerization of PANI and PPy to optimize the growth of these polymers and consequently ensure well-dispersed conductive polymers in the nanocellulose substrate. Acids that have often been used for such purposes include hydrochloric acid, sulfuric acid, and dodecylbenzenylsulfonic acid [54,63,64,65]. However, it is difficult to have a suitable solvent that could be suitable for most conductive polymers when using a coating method to fabricate high-performance conductive nanocellulose films. Neither is coating nor electrodeposition applicable for loading conductive polymers onto nanocellulose films, because of the thermal degradation of the polymers at the elevated temperature. As a result, in situ polymerization is becoming the most popular way to incorporate conductive polymers into nanocellulose hybrids [59,66]. However, the drawbacks of in situ polymerization include its complicated processes and environmental concerns: the time-consuming reactions involve multiple steps and toxic solvents.

**Figure 4:**
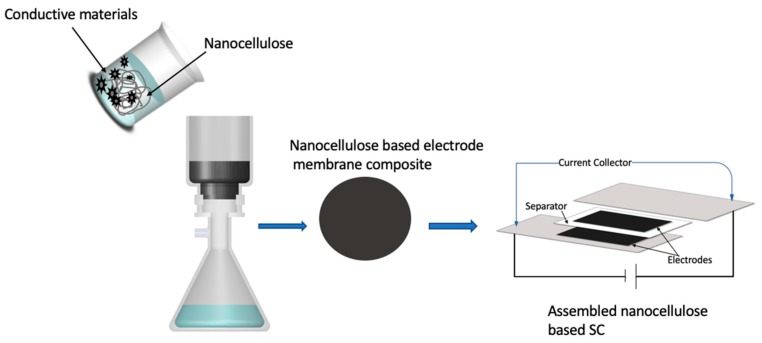
Filtration procedure to fabricate nanocellulose-based supercapacitor (SC).

Nanocellulose-based composite membrane electrodes can also be fabricated by a simple filtration system (Figure 4) [67,68,69,70] with conductive components incorporated via blending or in situ polymerization. Specifically, nanocellulose suspension and conductive component suspension or solvent can be mixed and subsequently transferred to a filtration system, in which the liquid(s) are allowed to pass through the filter and a well-mixed conductive materials/nanocellulose composite membrane is left behind on the filter. An air-dried composite membrane can be later peeled from the filter membrane for further use [67,68,69,70,71]. 

### 3.3. Nanocellulose Supercapacitors Loaded with Conductive Carbon Materials

Conductive carbon materials include single wall/multiple wall carbon nanotubes, graphene oxide, reduced graphene, and graphite. Conductive carbon materials generally have high conductivity and high tensile strength. Among them, carbon nanotubes have been known for their ultra-high tensile strength; graphene is a two-dimensional structure with an atomic thickness that offers extraordinary mechanical properties including a Young’s modulus of 1 TPa, and an ultimate strength of 130 GPa [57]. Carbon materials could be either coated onto the nanocellulose film surface or be incorporated into nanocellulose via blending. Blending usually allows more carbon particles to be incorporated into the nanocellulose substrate as compared to surface coating, due to the carbon particles possibly becoming entrapped inside the substrate, leading to an improved conductivity of the composite membrane [66,72]. Coating carbon nanomaterials onto nanocellulose membranes can also decrease transparency, which will consequently limit their applications in advanced photosensitive conductive materials. These specific hybrids showed an excellent conductivity and electromechanical stability when stretching and bending. A recent study demonstrated flex electrodes composed of graphene and conductive polymers-coated nanocellulose matrix, which showed a capacitance of 373 F/g at 1A/g and a cycling stability at 85% of capacitance retention after 1000 cycles [73]. 

### 3.4. Cellulose Membranes Loaded with Multiple Conductive Components

Each of the above-mentioned types of conductive material have advantages and limitations. Metallic particles have higher conductivity but lower capacity as compared to conductive polymers and carbon materials. Although carbon materials have high surface areas, superior chemical stability, extraordinary mechanical properties, and power density, they have the drawbacks of low specific capacitances and poor flexibility [74]. There are also concerns regarding the toxicity of some carbon materials such as carbon nanotubes, which are very popular ingredients for fabricating electrodes of energy storage devices. Conductive polymers can deliver as much as 10 times the energy, but have relatively lower electrical conductivity compared to carbon-based materials [9]. Conductive polymers are also not stable because of the pseudocapacitance reactions. Therefore, it has been a challenge to develop conductive materials with all the desirable properties for advanced energy devices. 

Several studies have reported the limitations of cycling stability, conductivity, or specific capacitance on nanocellulose-based SCs, which are loaded with a single type of conductive material [68,75,76,77]. Therefore, multiple conductive materials have been used in fabricating composite membrane electrodes to optimize the electrochemical performance of nanocellulose-based SCs and overcome the drawbacks of a single component. For example, carbon materials and conductive polymers can be blended together to improve both the stability and electrochemical performance of nanocellulose-based SCs. It has also been suggested that the electrochemical stability of PANI can be improved by incorporating reduced graphene oxide into PANI-based electrodes or supercapacitors [71,75,77,78]. This is because of the pseudocapacitance processes of PANI during charge–discharge cycles, in which part of the conductive polymerization product emeraldine PANI may convert to pernigraniline, which is a semiconductive polymer. This is because of the redox reactions of the ions transfer between two electrodes in pseudocapacitance polymers [76,79,80]. This situation involves the electrodes’ material deformation, including swelling, shrinkage, or cracking during charge and discharge cycles, which result in the poor working stability in the supercapacitors [65,68,70,81]. In addition, the degradation of PANI may be accelerated by the deformation of electrodes; thus, it can decrease the potential of working electrodes [9,65,68,70,81]. Therefore, the combination of other materials with PANI provides synergetic benefits regarding electrochemical performance. Metallic material (Ag nanowire, Au) or carbon materials (GO/RGO, carbon nanotubes) can improve conductivity [68,82] or decrease the V_IR_ drop on PANI-based supercapacitors. Therefore, the combination of multiple conductive materials can improve the overall electrochemical property [54,68,70,75,77,81]. Some studies reported that a synergistic effect on RGO incorporated with PANI fabricated a double-layer supercapacitor that raised the high pseudocapacitance and conductivity of the device [65,83,84,85]. 

Besides PANI, another conductive polymer polypyrrole (PPy) has similar limitations with PANI because of their similar electrochemical properties. Therefore, similar solutions can be used to overcome such limitations. Carbon materials or other metallic particles can be incorporated along with PPy to fabricate cellulose-based SCs or electrode membranes to achieve the expected performance [73,86,87]. On the other hand, some flexible electrode membranes were made by coating metallic particles on top of cellulose or printing papers. However, this type of electrode membrane or SC did not maintain good specific capacitance when compared to the SC made by conductive polymers [55,57,88]. Adding conductive polymers significantly improves the specific capacitance and stability of the hybrid electrodes after hundreds or thousands charge and discharge cycles [89,90,91,92]. 

A combination of different materials in a composite structure has been extensively studied as a strategy to obtain synergetic effects. Approaches for such combination include coating and blending [86,87,88,93,94,95,96,97], doping [57,98], in situ polymerization [62,65,86,87,94,99], and inkjet printing [55,100,101]. It is a challenge to control the thickness of the conductive layer by coating/blending and the in situ polymerization method. It is difficult to achieve a well-dispersed morphology of the conductive materials on the nanocellulose substrate. On the other hand, doping often results in a well-controlled thickness and well-dispensed morphology of the conductive layer, and therefore leads to an enhanced conductivity of the conductive hybrids’ membrane [102,103]. Inkjet printing can produce a layer of conductive materials with uniform thickness coated on top of the cellulose substrate, and results in conductive membranes with good mechanical strength and flexibility. However, the conductive materials are not well-dispersed into the cellulose substrate as compared to the blending method, which therefore may lead to cracking in the top layer of the printed conductive materials after thousands of running cycles [55,100,101].

On the other hand, solvent exchange and pressure extrusion are highly efficient ways to incorporate both metallic particles and conductive polymers to fabricate conductive membranes or energy storage devices. However, they have barely been applied on energy storage devices based on cellulose. Although solvent exchange is a rapid room-temperature process to synthesis conductive polymers without complicated procedures, cellulose may still get degraded in such high concentrated and toxic solvents [104], and therefore affect the mechanical performance of the cellulose-based conductive membranes. Pressure extrusion involves high energy consumption during the fabrication of conductive hybrids due to the requirement of high pressure (~3 ton) and temperature (over 160 °C) conditions [105,106], which may not be suitable for processing cellulose-based substrates. 

Overall, coating/blending, inkjet printing, and in situ polymerization are the most popular techniques that have been used to fabricate cellulose-based conductive membranes, because they involve simple procedures without advanced instruments or special conditions.

**Table 2:** Summary of nanocellulose-based conductive membranes developed for supercapacitors and energy storage devices.

Source of Nanocellulose	Conductive Materials	Methods	Highlights of Studies	Applications	References
Plants	SWCNTs	Inkjet printing	Ultra-strong, transparent, conductive, flexible, and printable nanocomposites	Supercapacitor	[107]
Plants	PANI/SWCNT	Solution deposition	Outstanding redox reaction, high energy density, and high power density	Supercapacitor	[62]
Plants	PANI	Coating	Flexible electrodes without binder	Supercapacitor	[108]
Plants	CNT/PANI	Lay-by-lay coating	High flexibility and good cycling stability, high-loading and high-energy capacitance	Wearable portable devices	[60]
Plants/Bacterial	PPy/GO	Coating/in situ polymerization	Free standing, light weight, low cost, high conductive performance and capacitance	Paper electrodes	[73,86,87]
Plants	PPy/GO	In situ polymerization, blending	Flexible, very high volumetric capacitance	Paper electrodes	[95]
Plants	Indium oxide, CNT, Silver nanowires	N-Doped	Highly transparent, improved optical property	Solar cell, touch screen, interactive paper	[57]
Plants	Nickel-phosphorus (Ni-P)	Coating	Feasible to control the crystallite size and hollow cavity pore size	Electrodes	[88]
Plants	PANI	In situ polymerization	Improved capacitance, good electrochemical performance after 1000 cycles	All-solid-state supercapacitor	[65]
Plants	Graphite foil, graphene, nanographite,	Coating	Low-cost and eco-friendly aqueous supercapacitor	Supercapacitor, water-purification carbons	[93]
Wood	GO/graphene	Hybrid/coating/blending	Large contact area	Li-S batteries	[96]
Softwood	Silver nanoparticle ink	Inkjet printing	High optical transparency, sufficiently high performance, low coefficient of thermal expansion	Paper device	[55]
Paper	Active carbon/CNT, Ag Nanowires	Inkjet printing	User-customized control of cell, free-standing flexible supercapacitor	Supercapacitor	[100]
Filter paper	Ni/active carbon, Ni/MnO_2_	Electroless plating, Electrodeposition	A free-standing flexible asymmetrically supercapacitor, large volume density, and superior flexibility	Supercapacitor	[109]
Paper/PVA	PANI/Au	In situ depositing	Flexible self-powered piezoelectric generator	Supercapacitor	[82]
Cellulose	MWCNT	Doping	Excellent tensile test result, high Young’s modulus	Supercapacitor	[98]
Cellulose	rGO/PANI	Coating, in situ polymerization	Highly flexible/foldable, high electrochemical performance	Supercapacitor	[110]
Bacterial cellulose	PANI	In situ depositing	New method of peeling off conductive film	Supercapacitor	[111]
Bacterial cellulose	PPy/copper oxide	Coating	Free-standing, flexible	Supercapacitor	[48]
Bacterial cellulose	CNT/GO/RGO	In situ polymerization	Excellent capability, cycling stability	Electrodes	[73,99]
Bacterial cellulose	SiNPs/PANI	Binding/coating	Stable, flexible, less stress dissipation	Electrodes	[97]
Bacterial cellulose	PPy	In situ polymerization. coating	Flexible, high electrical/electrochemical performance	Electroactive membrane	[94]

## 4. Evaluation of Electrical Performance of Nanocellulose-Based Supercapacitors

The specific capacitance, operating voltage window, equivalent series resistance (ESR), and power density/energy density are key parameters to evaluate the electrical performance of all types of supercapacitors, including nanocellulose-based SCs [11,112]. Life cycles and stability are also important factors that need to be considered when evaluating SCs [9,113,114,115]. The galvenostatic charge–discharge cycle (GDC) test is widely used for the characterization of electrochemical performance among the assembled supercapacitors [11,112,116,117]. It is a time period between charging and discharging when a constant peak voltage remains. An internal resistance voltage drop (V_IR_) appears during switching between charging and discharging, which is an important parameter to evaluate the electrochemical performance of an SC. Key parameters for evaluating SCs other than those mentioned above can also be estimated from the GDC plot, and can be influenced by V_IR_. A large V_IR_ drop means that a large amount of energy is being wasted during charge–discharge cycles, which leads to a large resistance and the overall low performance of the SCs [115]. An increase in the loading ratio of conductive materials in a nanocellulose-based SC can generally contribute to a lower V_IR_ drop and better electrochemical performance [5,9,115]. One study developed a nanocellulose-based SC with excellent conductivity and electrochemical performance by combining graphene and conductive polymers together to increase the active mass ratios up to 94% [118]. A high current is needed to achieve a specific high current density if the electrodes contain a high mass loading ratio of conductive materials, which may lead to a larger V_IR_ drop when a high current is applied. As a result, both the current density and the mass loading ratio should be considered when comparing gravimetric current density data between electrodes developed in different work [9]. 

To enable comparison of the specific capacitance, power density, and energy density between different nanocellulose membrane SCs, data on electrochemical performance per unit area, weight, or volume are usually used. Some examples are the specific capacitance (F cm^−2^, F Kg^−1^, F cm^−3^), specific power density (W cm^−2^, W kg^−1^, or W cm^−3^), and specific energy density (Wh kg^−1^, Wh cm^−2^, or Wh cm^−3^) [11,112]. However, both two-electrode systems and three-electrode systems in a closed circuit during running charge–discharge tests have been seen in similar studies, making it difficult to make a comparison among these studies [66]. Other details in the design of membrane electrodes or SCs, such as the types of current collectors, separators, packaging materials, charge–discharge current, and voltage scan rate, can also affect their electrochemical performance and make it difficult to compare the materials that have been developed in different reports. The gravimetric capacitance and areal capacitance are two key benchmarks that are often used to compare materials and evaluate the electrochemical properties of energy storage devices. Still, the thickness of an electrode can impact the evaluation results on area-based capacitances. Some studies reported a high specific capacity in a nanocellulose-based SC based on the weight of a thin layer of the electroactive material. Still, the total weight of the electrode as a whole should be considered [9,119]. Furthermore, particularly for the electrodes containing porous substrates such as nanocellulose, the weight of electrolytes may also be taken into account when the gravimetric capacitance is calculated [5,9,115]. The weight of the electrolytes presented in the nanocellulose-based electrodes needs to be minimized in order to increase the calculated specific capacitance [5,9,115]. 

In addition to electrochemical evaluations, mechanical tests such as tensile or bending tests are also important for nanocellulose-based supercapacitors. A large amount of conductive materials loaded onto the nanocellulose substrate can make the electrode membranes brittle and easy to crack when working. However, only a few recent studies on nanocellulose-based SCs showed mechanical test results [65,82,110]. Therefore, a lot more data is needed on the mechanical evaluations of nanocellulose-based SCs to provide a full picture of the overall performance of nanocellulose-based SCs. 

## 5. Applications of Nanocellulose-Based Electronic Devices

With the fast growth of the world market for sensors, a variety of sensors are highly demanded for environment pollution prevention, health management, and biomedical applications. In particular, nanocellulose-based sensors for health management and biomedical applications are attracting research interest [120]. Nanocellulose, as a great flexible and ultra-thin substrate, can support electronic components to fabricate biomedical sensors to be integrated onto or into the human body [121,122]. Nanocellulose-based biomedical sensors for home uses such as glucose and cholesterol sensors can help patients better manage their chronic diseases. Some other biomarkers including gases, proteins, DNA, pathogens, or toxic compounds can also be detected by nanocellulose-based sensors [121,122]. A recent study used functionalized cellulose nanocrystals coated with polyvinylpyrrolidone to fabricate a conductive film as a biosensor that can distinguish similar chemical compounds [121]. The highly sensitive cellulose-based sensor showed great potential for detecting biomolecules in the human body. Another study fabricated a conductive film with a TEMPO-oxidized nanocellulose hydrogel based on graphene quantum dots to monitor the fluorescent quenching that can be used for laccase enzymes detection [123]. This nanocellulose-based sensor can increase the signal regarding the intensity of the fluorescent without shifting the wavelengths [123]. Besides, a TEMP-oxidized nanocellulosed biosensor can be used to detect biomolecules, C-phycocyanin, and copper ions [124]. 

Nanocellulose-based solar cells as photoelectric conversion devices can provide high power conversion efficiency with low cost, and have subsequently become one of the most promising devices to replace conventional solar cells [125,126]. Most flexible solar cells films are made by polyethylene terephthalate (PET) and polyethylene naphthalate (PEN), which are not easily degraded and can lead to serious environmental concerns [125,126]. The nanocellulose-based solar cells with excellent optic transparency and flexibility can be widely applied in wearable electronics to fill up the rapidly increasing demand for consumer electronics. A recent study developed a nanocellulose-based flexible perovskite solar cell by coating PEDOT:PSS doped with Triton-X 100 and ethylene glycol [127]. This transparent solar cell can retain over 80% of energy storage efficiency after 50 times of bending. It shows great potential for transparent conductive electrodes, touch screens, and transistors [127]. Another recent study developed a nanocellulose-based magnetic core–shell with titanium chloride composite as a dye-sensitive solar cell. It showed a high capacitance at 14.90 mA cm^-2^ and energy conversion efficiency at 64% [128]. 

A nanocellulose-base paper battery has recently attracted attention in relation to smart and wearable customers’ electronic devices [129,130]. The electrochemical performance of the nanocellulose-based paper battery can be comparable to that of a conventional battery, and it is an eco-friendly and degradable electronic device that shows a great potential to replace the conventional batteries [129,130,131]. Normally, the conventional battery electrodes usually consist of a polymer binder of electrodes such as polyvinylidene fluoride (PVdF), which requires a toxic organic solvent such as N-methyl-2-pyrrolidinone (NMP) during fabrication processes [129,130,131]. On the other hand, a nanocellulose-based paper battery doesn’t require complicated process and hazardous solvents. Wang et al. reported on a nanocellulose structured paper-based lithium battery [132]. Nanocellulose provides a 3D porous structure incorporated with carbon nanofibers and LiFePO_4_ that can maintain an excellent cycling capacity (85%) even after 1000 cycles [132]. Another recent study used nanocellulose-modified polyethylene separators in the lithium battery to improve the cycling stability of a high-energy density lithium battery [133]. The nanofiber layer is thermally stable and flexible with hydrophilic property; it was coated with polyethylene on both sides to fabricate a novel trilayer separator that significantly enhanced the cycling stability and safety of the lithium battery [133]. Those recent studies showed that nanocellulose-based batteries have great potential to replace the conventional ones and solve the stability and safety concerns of lithium batteries. 

In addition to the nanocellulose-based electronic devices that have been mentioned above, other applications include E-skin, electric vehicles, fuel cells, military backup power, and some smart consumer electronic devices. Among those applications for nanocellulose-based electronic devices, supercapacitors are a major application that can be widely applied in manufactures. However, most of the studies only provide evidence and data at the laboratory level to show the potential of nanocellulose for those applications. Thus, more research and development efforts are needed to transfer the studies from labs to manufacturing. In fact, there are still a lot of challenges that need to be conquered in order to meet the requirements of the market. 

## 6. Challenges of Nanocellulose-Based Supercapacitors 

Nanocellulose-based SCs are sustainable and cost-effective energy storage devices that can exhibit a large specific capacitance and a stable, long cycling life. However, the data regarding the evaluation of their electrochemical performance in different reports are hardly comparable unless key processing factors are taken into account, as discussed in Section 4, which may be a challenge in the research and development efforts of nanocellulose-based SCs.

Another challenge for the development of nanocellulose-based supercapacitors is to optimize different aspects of the performances of the materials, specifically the mechanical performance and the electrochemical performance, which are sometimes contradictory. For example, the thickness of the electrodes and the thickness of the coating layer on the nanocellulose substrate are very important factors for the electrochemical performance. The thickness of the active materials coated on the nanocellulose substrate ranges from nanometers up to micrometers, which contribute to a small part of the whole electrodes. Although the gravimetric specific capacitance of the active material is high, it gives a low volumetric capacitance in the nanocellulose-based electrodes because of the generally low active mass ratio in the nanocellulose composites [9,115,134]. This problem cannot actually be solved by increasing the thickness of the coating layer, since the low efficiency in ions transport results in poor capabilities and relatively low volumetric capacitances [9,115,134]. In order to solve the issue, a type of nanocellulose-based electrode matrix was fabricated by blending the active materials into the nanocellulose substrate. This method can blend several conductive materials and nanocellulose substrates together to form lightweight, mechanically flexible electrodes [96,97,119]. However, most conductive materials are heavy and brittle. An overloaded nanocellulose substrate may decrease in mechanical strength. It is a big challenge to develop a nanocellulose-based SC that has not only strong mechanical strength but also a high loading of active materials to achieve high energy density. It has been suggested in several previous studies that loading conductive materials into the inner pores of the nanocellulose substrate may increase the conductive mass ratio and consequently may improve the energy density as well as maintain good mechanical strength [96,97,119]. 

Generally, the higher the weight ratio of conductive materials in the electrode, the more energy can be stored in the device, which results in a larger areal capacitance [81,135]. This is because the nanocellulose-based SC made by the blending method has extremely high porosities that improve the ions’ transportation rate and increase the conductivity. However, its low volumetric capacitance is still a big challenge to be solved, because low volumetric density will increase the weight ratio of the electrolyte inside the pores [5,9,115]. Another type of flexible nanocellulose-based SC has been developed by coating conductive polymers on the surface of the individual fibers. This method is particularly interesting, mainly because the procedures are relatively straightforward and inexpensive [65,73,86,87,94,99,111]. This type of SC had higher active mass loadings than other types of nanocellulose-based electrodes. The conductive materials in this type of electrodes ranged from 25% to more than 75% and minimized the weight of the electrolytes. The advantage of this type of electrode is the maximum utilization rate of conductive materials and the increase in the efficiency in energy storage and release ability, even though the thickness of electrodes can be hundreds of micrometers. Although the conductivity has been significantly improved by coating conductive polymers onto individual fibers, the heavy mass content may bring down current density, which will further lower down the power density and energy density. Moreover, the mechanical performance may be decreased by coating the conductive agents onto individual fibers, because the conductive polymers are heavy and brittle, and a single fiber may not withstand high mechanical stress. 

Considering the advantages and disadvantages of different methods that are used to fabricate nanocellulose-based supercapacitors, there are still a lot of questions and challenges to be solved in order to obtain excellent properties both electrochemically and mechanically. 

## 7. Summary

Cellulose nanofibers are an abundant polymer in nature. Due to its excellent mechanical properties, it has been considered as a potential biomaterial for a broad range of applications, including energy storage. Nanocellulose materials have a high surface area to volume ratio, high crystallinity, and high transparency, which are needed for such applications. Generally, nanofibers can be prepared by several methods, such as high-pressure homogenizer, electrospinning, steam, and cryocrushing. Pre-treatments could help reduce the energy consumption during the isolation processes. Although the pre-treatments accelerated the productivity of nanofibers, the current concern is to improve the mechanical properties after the pre-treatments.

Nanocellulose-based SCs have been fabricated by loading various conductive components—including metallic particles, carbon materials, and conductive polymers—into the nanocellulose substrate. Each type of conductive material has its own advantages and drawbacks. As a result, multiple conductive materials have usually been loaded in combination onto nanocellulose substrates in recent studies to provide synergistic results in the designs. Therefore, it is crucial to find appropriate ratios between different the components of the designed supercapacitors to ensure an optimized overall performance in both electrochemical and mechanical aspects. Coating/blending, inkjet printing, and in situ polymerization have been the most popular methods for fabricating nanocellulose-based conductive membranes for free-standing supercapacitors and energy storage devices. Each method for fabricating nanocellulose-based SCs has its own advantages and disadvantages. There are still many challenges to be overcome before such highly promising materials can be transferred from labs to a wide variety of real-life applications.

## Figures and Tables

**Table 1:** The comparison of properties of natural cellulose, carbon, and E-glass fibers [3,18].

Fiber	Density (g/cm^3^)	Diameter (µm)	Tensile Strength (MPa)	Young’s Modulus (GPa)	Elongation at Break (%)
Hemp	1.47	25–50	690	70	1.6
Kenaf	1.49	20–60	930	53	1.6
Sisal	1.5	30–50	467–700	9.4–22	3–7
Cotton	1.5	12–38	287–800	5.5–12.6	7–8
Oil Palm	0.7–1.55	15–50	248	3.2	25
Carbon	1.78	5–7	3400–4800	240	1.4–1.8
E-glass	2.55	<17	3400	73	2.5

## References

[1] Wegner T.H., Jones P.E. (2006). Advancing cellulose-based nanotechnology. Cellulose.

[2] Deepa B., Abraham E., Cherian B.M., Bismarck A., Blaker J.J., Pothan L.A., Leao A.L., De Souza S.F., Kottaisamy M. (2011). Structure, morphology and thermal characteristics of banana nano fibers obtained by steam explosion. Bioresour. Technol..

[3] Mohanty A., Misra M., Hinrichsen G. (2000). Biofibres, biodegradable polymers and biocomposites: An overview. Macromol. Mater. Eng..

[4] Wang G., Zhang L., Zhang J. (2012). A review of electrode materials for electrochemical supercapacitors. Chem. Soc. Rev..

[5] Yu D.S., Qian Q.H., Wei L., Jiang W.C., Goh K.L., Wei J., Zhang J., Chen Y. (2015). Emergence of fiber supercapacitors. Chem. Soc. Rev..

[6] Beidaghi M., Gogotsi Y. (2014). Capacitive energy storage in micro-scale devices: Recent advances in design and fabrication of micro-supercapacitors. Energy Environ. Sci..

[7] Conway B.E. (1991). Transition from “supercapacitor” to “battery” behavior in electrochemical energy storage. J. Electrochem. Soc..

[8] Zhu H., Luo W., Ciesielski P.N., Fang Z., Zhu J.Y., Henriksson G., Himmel M.E., Hu L. (2016). Wood-Derived Materials for Green Electronics, Biological Devices, and Energy Applications. Chem. Rev..

[9] Wang Z.H., Tammela P., Stromme M., Nyholm L. (2017). Cellulose-based Supercapacitors: Material and Performance Considerations. Adv. Energy Mater..

[10] Conway B., Birss V., Wojtowicz J. (1997). The role and utilization of pseudocapacitance for energy storage by supercapacitors. J. Power Sour..

[11] Supercapacitors E., Conway B.E. (1999). Scientific Fundamentals and Technological Applications.

[12] Zheng W., Lv R., Na B., Liu H., Jin T., Yuan D. (2017). Nanocellulose-mediated hybrid polyaniline electrodes for high performance flexible supercapacitors. J. Mater. Chem. A.

[13] Cai J., Niu H., Wang H., Shao H., Fang J., He J., Xiong H., Ma C., Lin T. (2016). High-performance supercapacitor electrode from cellulose-derived, inter-bonded carbon nanofibers. J. Power Sour..

[14] Islam N., Li S., Ren G., Zu Y., Warzywoda J., Wang S., Fan Z. (2017). High-frequency electrochemical capacitors based on plasma pyrolyzed bacterial cellulose aerogel for current ripple filtering and pulse energy storage. Nano Energy.

[15] Li Z., Liu J., Jiang K., Thundat T. (2016). Carbonized nanocellulose sustainably boosts the performance of activated carbon in ionic liquid supercapacitors. Nano Energy.

[16] Ruan C.-Q., Wang Z., Lindh J., Strømme M. (2018). Carbonized cellulose beads for efficient capacitive energy storage. Cellulose.

[17] Liu Y., Chen J., Cui B., Yin P., Zhang C. (2018). Design and preparation of biomass-derived carbon materials for supercapacitors: A review. C.

[18] Nizamuddin S., Siddiqui M., Mubarak N., Baloch H.A., Mazari S.A., Tunio M., Griffin G., Srinivasan M., Tanksale A., Riaz S. (2018). Advanced nanomaterials synthesis from pyrolysis and hydrothermal carbonization: A review. Curr. Org. Chem..

[19] Jonoobi M., Oladi R., Davoudpour Y., Oksman K., Dufresne A., Hamzeh Y., Davoodi R. (2015). Different preparation methods and properties of nanostructured cellulose from various natural resources and residues: A review. Cellulose.

[20] Siqueira G., Bras J., Dufresne A. (2010). Cellulosic bionanocomposites: A review of preparation, properties and applications. Polymers.

[21] Behzad T., Ahmadi M. (2016). Nanofibers. Nanofiber Research-Reaching New Heights.

[22] Islam M.T., Alam M.M., Zoccola M. (2013). Review on modification of nanocellulose for application in composites. Int. J. Innov. Res. Sci. Eng. Technol..

[23] Wang B., Sain M., Oksman K. (2007). Study of structural morphology of hemp fiber from the micro to the nanoscale. Appl. Compos. Mater..

[24] Habibi Y., Lucia L.A., Rojas O.J. (2010). Cellulose nanocrystals: Chemistry, self-assembly, and applications. Chem. Rev..

[25] Timell T.E. (1964). Wood hemicelluloses: Part I. Adv. Carbohydr. Chem..

[26] Chirayil C.J., Mathew L., Thomas S. (2014). Review of recent research in nano cellulose preparation from different lignocellulosic fibers. Rev. Adv. Mater. Sci..

[27] Harada H., Goto T. (1982). The structure of cellulose microfibrils in Valonia. Cellulose and Other Natural Polymer Systems.

[28] Ray D.P., Banerjee P., Satya P., Mitra S., Ghosh R.K., Mondal S.B. (2014). Degumming of decorticated ramie fibre through novel chemical process. Indian J. Nat. Fibers.

[29] Münder F., Fürll C., Hempel H. (2004). Advanced decortication technology for unretted bast fibres. J. Nat. Fibers.

[30] Paridah M.T., Basher A.B., SaifulAzry S., Ahmed Z. (2011). Retting process of some bast plant fibres and its effect on fibre quality: A review. BioResources.

[31] Ramamoorthy S.K., Skrifvars M., Persson A. (2015). A review of natural fibers used in biocomposites: Plant, animal and regenerated cellulose fibers. Polym. Rev..

[32] Shah D.U. (2013). Developing plant fibre composites for structural applications by optimising composite parameters: A critical review. J. Mater. Sci..

[33] Biagiotti J., Puglia D., Kenny J.M. (2004). A review on natural fibre-based composites-part I: Structure, processing and properties of vegetable fibres. J. Nat. Fibers.

[34] Mwaikambo L. (2006). Review of the history, properties and application of plant fibres. Afr. J. Sci. Technol..

[35] Pääkkö M., Ankerfors M., Kosonen H., Nykänen A., Ahola S., Österberg M., Ruokolainen J., Laine J., Larsson P.T., Ikkala O. (2007). Enzymatic hydrolysis combined with mechanical shearing and high-pressure homogenization for nanoscale cellulose fibrils and strong gels. Biomacromolecules.

[36] Stenstad P., Andresen M., Tanem B.S., Stenius P. (2008). Chemical surface modifications of microfibrillated cellulose. Cellulose.

[37] Chakraborty A., Sain M., Kortschot M. (2005). Cellulose microfibrils: A novel method of preparation using high shear refining and cryocrushing. Holzforschung.

[38] Wang B., Sain M. (2007). Dispersion of soybean stock-based nanofiber in a plastic matrix. Polym. Int..

[39] Joonobi M., Harun J., Tahir P.M., Zaini L.H., SaifulAzry S., Makinejad M.D. (2010). Characteristic of nanofibers extracted from kenaf core. BioResources.

[40] Habibi Y., Vignon M.R. (2008). Optimization of cellouronic acid synthesis by TEMPO-mediated oxidation of cellulose III from sugar beet pulp. Cellulose.

[41] Saito T., Hirota M., Tamura N., Kimura S., Fukuzumi H., Heux L., Isogai A. (2009). Individualization of nano-sized plant cellulose fibrils by direct surface carboxylation using TEMPO catalyst under neutral conditions. Biomacromolecules.

[42] Alila S., Besbes I., Vilar M.R., Mutjé P., Boufi S. (2013). Non-woody plants as raw materials for production of microfibrillated cellulose (MFC): A comparative study. Ind. Crop. Prod..

[43] Janardhnan S., Sain M.M. (2007). Isolation of cellulose microfibrils–an enzymatic approach. Bioresources.

[44] Henriksson M., Henriksson G., Berglund L., Lindström T. (2007). An environmentally friendly method for enzyme-assisted preparation of microfibrillated cellulose (MFC) nanofibers. Eur. Polym. J..

[45] Khalil H.A., Davoudpour Y., Islam M.N., Mustapha A., Sudesh K., Dungani R., Jawaid M. (2014). Production and modification of nanofibrillated cellulose using various mechanical processes: A review. Carbohydr. Polym..

[46] Miloh T., Spivak B., Yarin A. (2009). Needleless electrospinning: Electrically driven instability and multiple jetting from the free surface of a spherical liquid layer. J. Appl. Phys..

[47] Takagi H., Nakagaito A., Nishimura K., Matsui T. (2016). Mechanical characterisation of nanocellulose composites after structural modification. High Perform. Optim. Des. Struct. Mater. II.

[48] Du X., Zhang Z., Liu W., Deng Y. (2017). Nanocellulose-based conductive materials and their emerging applications in energy devices—A review. Nano Energy.

[49] Peng S., Fan L., Rao W., Bai Z., Xu W., Xu J. (2017). Bacterial cellulose membranes coated by polypyrrole/copper oxide as flexible supercapacitor electrodes. J. Mater. Sci..

[50] Wang X., Chen Y., Schmidt O.G., Yan C. (2016). Engineered nanomembranes for smart energy storage devices. Chem. Soc. Rev..

[51] Yang Y., Yu D., Wang H., Guo L. (2017). Smart Electrochemical Energy Storage Devices with Self-Protection and Self-Adaptation Abilities. Adv. Mater..

[52] Huang Y., Zhu M., Huang Y., Pei Z., Li H., Wang Z., Xue Q., Zhi C. (2016). Multifunctional energy storage and conversion devices. Adv. Mater..

[53] Maitra A., Karan S.K., Paria S., Das A.K., Bera R., Halder L., Si S.K., Bera A., Khatua B.B. (2017). Fast charging self-powered wearable and flexible asymmetric supercapacitor power cell with fish swim bladder as an efficient natural bio-piezoelectric separator. Nano Energy.

[54] Dubal D.P., Chodankar N.R., Kim D.-H., Gomez-Romero P. (2018). Towards flexible solid-state supercapacitors for smart and wearable electronics. Chem. Soc. Rev..

[55] Tan Y.T., Zhang Y.F., Kong L.B., Kang L., Ran F. (2017). Nano-Au@PANI core-shell nanoparticles via in-situ polymerization as electrode for supercapacitor. J. Alloy. Compd..

[56] Tan Y.T., Liu Y.S., Kong L.B., Kang L., Xu C.G., Ran F. (2017). In Situ doping of PANI nanocomposites by gold nanoparticles for high-performance electrochemical energy storage. J. Appl. Polym. Sci..

[57] Yagyu H., Saito T., Isogai A., Koga H., Nogi M. (2015). Chemical modification of cellulose nanofibers for the production of highly thermal resistant and optically transparent nanopaper for paper devices. ACS Appl. Mater. Interfaces.

[58] Ma X.W., Li Y., Wen Z.W., Gao F.X., Liang C.Y., Che R.C. (2015). Ultrathin beta-Ni(OH)(2) Nanoplates Vertically Grown on Nickel-Coated Carbon Nanotubes as High-Performance Pseudocapacitor Electrode Materials. ACS Appl. Mater. Interfaces.

[59] Hu L.B., Zheng G.Y., Yao J., Liu N.A., Weil B., Eskilsson M., Karabulut E., Ruan Z.C., Fan S.H., Bloking J.T. (2013). Transparent and conductive paper from nanocellulose fibers. Energy Environ. Sci..

[60] Chiang C.K., Fincher C., Park Y.W., Heeger A.J., Shirakawa H., Louis E.J., Gau S.C., MacDiarmid A.G. (1977). Electrical conductivity in doped polyacetylene. Phys. Rev. Lett..

[61] Eftekhari A. (2011). Nanostructured Conductive Polymers.

[62] Dong L.B., Liang G.M., Xu C.J., Ren D.Y., Wang J.J., Pan Z.Z., Li B.H., Kang F.Y., Yang Q.H. (2017). Stacking up layers of polyaniline/carbon nanotube networks inside papers as highly flexible electrodes with large areal capacitance and superior rate capability. J. Mater. Chem. A.

[63] Fan Z.M., Cheng Z.J., Feng J.Y., Xie Z.M., Liu Y.Y., Wang Y.S. (2017). Ultrahigh volumetric performance of a freestanding compact N-doped holey graphene/PANI slice for supercapacitors. J. Mater. Chem. A.

[64] Jiang Q.S., Kacica C., Soundappan T., Liu K.K., Tadepalli S., Biswas P., Singamaneni S. (2017). An in situ grown bacterial nanocellulose/graphene oxide composite for flexible supercapacitors. J. Mater. Chem. A.

[65] Lv X.D., Li G.H., Li D.W., Huang F.L., Liu W.T., Wei Q.F. (2017). A new method to prepare no-binder, integral electrodes-separator, asymmetric all-solid-state flexible supercapacitor derived from bacterial cellulose. J. Phys. Chem. Solids.

[66] Feng E.K., Peng H., Zhang Z.G., Li J.D., Lei Z.Q. (2017). Polyaniline-based carbon nanospheres and redox mediator doped robust gel films lead to high performance foldable solid-state supercapacitors. New J. Chem..

[67] Liu F.W., Luo S.J., Liu D., Chen W., Huang Y., Dong L., Wang L. (2017). Facile Processing of Free-Standing Polyaniline/SWCNT Film as an Integrated Electrode for Flexible Supercapacitor Application. ACS Appl. Mater. Interfaces.

[68] Zhang X., Wu X., Lu C., Zhou Z. (2015). Dialysis-free and in situ doping synthesis of polypyrrole@ cellulose nanowhiskers nanohybrid for preparation of conductive nanocomposites with enhanced properties. ACS Sustain. Chem. Eng..

[69] Khosrozadeh A., Darabi M.A., Xing M., Wang Q. (2015). Flexible Cellulose-Based Films of Polyaniline–Graphene–Silver Nanowire for High-Performance Supercapacitors. J. Nanotechnol. Eng. Med..

[70] Khosrozadeh A., Darabi M.A., Xing M., Wang Q. (2016). Flexible electrode design: Fabrication of freestanding polyaniline-based composite films for high-performance supercapacitors. ACS Appl. Mater. Interfaces.

[71] Khosrozadeh A., Xing M., Wang Q. (2015). A high-capacitance solid-state supercapacitor based on free-standing film of polyaniline and carbon particles. Appl. Energy.

[72] Hsu H.H., Khosrozadeh A., Li B., Luo G., Xing M.M., Zhong W. (2019). An Eco-friendly, Nanocellulose/RGO/in-situ Formed Polyaniline for Flexible and Free-standing Supercapacitors. ACS Sustain. Chem. Eng..

[73] Liang H.-W., Guan Q.-F., Song L.-T., Yao H.-B., Lei X., Yu S.-H. (2012). Highly conductive and stretchable conductors fabricated from bacterial cellulose. NPG Asia Mater..

[74] Luan D.X., Zhang X.W., Yu Y., Chen Y.H., Ma Y., Bi C.L., Zhao D.Y. (2017). Fabrication and electrochemical properties of graphene/copper-nickel solid solution reinforced polyaniline composite. J. Mater. Sci.-Mater. Electron..

[75] Kang Z.P., Jiao K.L., Xu X.P., Peng R.Y., Jiao S.Q., Hu Z.Q. (2017). Graphene oxide-supported carbon nanofiber-like network derived from polyaniline: A novel composite for enhanced glucose oxidase bioelectrode performance. Biosens. Bioelectron..

[76] Snauwaert P., Lazzaroni R., Riga J., Verbist J., Gonbeau D. (1990). A photoelectron spectroscopic study of the electrochemical processes in polyaniline. J. Chem. Phys..

[77] Manoj M., Anilkumar K.M., Jinisha B., Jayalekshmi S. (2017). Polyaniline-Graphene Oxide based ordered nanocomposite electrodes for high-performance supercapacitor applications. J. Mater. Sci.-Mater. Electron..

[78] Shabani-Nooshabadi M., Zahedi F. (2017). Electrochemical reduced graphene oxide-polyaniline as effective nanocomposite film for high-performance supercapacitor applications. Electrochim. Acta.

[79] Ćirić-Marjanović G. (2013). Recent advances in polyaniline research: Polymerization mechanisms, structural aspects, properties and applications. Synth. Metals.

[80] Kulkarni V.G., Campbell L.D., Mathew W.R. (1989). Thermal stability of polyaniline. Synth. Metals.

[81] Wang H., Lin J., Shen Z.X. (2016). Polyaniline (PANi) based electrode materials for energy storage and conversion. J. Sci. Adv. Mater. Devices.

[82] Yuan L., Xiao X., Ding T., Zhong J., Zhang X., Shen Y., Hu B., Huang Y., Zhou J., Wang Z.L. (2012). Paper-Based Supercapacitors for Self-Powered Nanosystems. Angew. Chem..

[83] Aytug T., Rager M.S., Higgins W., Brown F.G., Veith G.M., Rouleau C.M., Wang H., Hood Z.D., Mahurin S.M., Mayes R.T. (2018). Vacuum-Assisted Low-temperature Synthesis of Reduced Graphene Oxide Thin Film Electrodes for High Performance Transparent and Flexible All-Solid-State Supercapacitors. ACS Appl. Mater. Interfaces.

[84] Hu R., Zhao J., Zhu G., Zheng J. (2018). Fabrication of flexible free-standing reduced graphene oxide/polyaniline nanocomposite film for all-solid-state flexible supercapacitor. Electrochim. Acta.

[85] Lin Y., Zhang H., Deng W., Zhang D., Li N., Wu Q., He C. (2018). In-situ growth of high-performance all-solid-state electrode for flexible supercapacitors based on carbon woven fabric/polyaniline/graphene composite. J. Power Sour..

[86] Carlsson D.O., Sjodin M., Nyholm L., Stromme M. (2013). A Comparative Study of the Effects of Rinsing and Aging of Polypyrrole/Nanocellulose Composites on Their Electrochemical Properties. J. Phys. Chem. B.

[87] Wang Z.H., Tammela P., Zhang P., Stromme M., Nyholm L. (2014). Efficient high active mass paper-based energy-storage devices containing free-standing additive-less polypyrrole-nanocellulose electrodes. J. Mater. Chem. A.

[88] Pan Y.F., Guo Z.Q., Guo T.C., Wang X., Huang J.T. (2016). The preparation, characterization, and influence of multiple electroless nickel-phosphorus (Ni-P) hollow composite coatings on micro-nano cellulose fibers. Surf. Coat. Technol..

[89] Du P.C., Lin L., Wang H.X., Liu D., Wei W.L., Li J.G., Liu P. (2017). Fabrication of porous polyaniline modified MWNTs core-shell structure for high performance supercapacitors with high rate capability. Mater. Des..

[90] Muralikrishna S., Nagaraju D.H., Balakrishna R.G., Surareungchai W., Ramakrishnappa T., Shivanandareddy A.B. (2017). Hydrogels of polyaniline with graphene oxide for highly sensitive electrochemical determination of lead ions. Anal. Chim. Acta.

[91] Wang B., Liu X., Liu Q.Z., Chen J.H., Jiang H.Q., Wang Y.D., Liu K., Li M.F., Wang D. (2017). Three-dimensional non-woven poly(vinyl alcohol-co-ethylene) nanofiber based polyaniline flexible electrode for high performance supercapacitor. J. Alloy. Compd..

[92] Wang H.X., Liu D., Du P.C., Wei W.L., Wang Q., Liu P. (2017). Comparative study on polyvinyl chloride film as flexible substrate for preparing free-standing polyaniline-based composite electrodes for supercapacitors. J. Coll. Interface Sci..

[93] Blomquist N., Wells T., Andres B., Backstrom J., Forsberg S., Olin H. (2017). Metal-free supercapacitor with aqueous electrolyte and low-cost carbon materials. Sci. Rep..

[94] Lay M., Gonzalez I., Tarres J.A., Pellicer N., Bun K.N., Vilaseca F. (2017). High electrical and electrochemical properties in bacterial cellulose/polypyrrole membranes. Eur. Polym. J..

[95] Wang Z.H., Tammela P., Stromme M., Nyholm L. (2015). Nanocellulose coupled flexible polypyrrole@graphene oxide composite paper electrodes with high volumetric capacitance. Nanoscale.

[96] Patel M.U.M., Luong N.D., Seppala J., Tchernychova E., Dominko R. (2014). Low surface area graphene/cellulose composite as a host matrix for lithium sulphur batteries. J. Power Sour..

[97] Park M., Lee D., Shin S., Kim H.J., Hyun J. (2016). Flexible conductive nanocellulose combined with silicon nanoparticles and polyaniline. Carbohydr. Polym..

[98] Zhang X., Lin Z., Chen B., Sharma S., Wong C.-p., Zhang W., Deng Y. (2013). Solid-state, flexible, high strength paper-based supercapacitors. J. Mater. Chem. A.

[99] Chang Y.H., Zhou L., Xiao Z.C., Liang J.X., Kong D.B., Li Z.H., Zhang X.H., Li X.L., Zhi L.J. (2017). Embedding Reduced Graphene Oxide in Bacterial Cellulose-Derived Carbon Nanofibril Networks for Supercapacitors. Chemelectrochem.

[100] Choi K.-H., Yoo J., Lee C.K., Lee S.-Y. (2016). All-inkjet-printed, solid-state flexible supercapacitors on paper. Energy Environ. Sci..

[101] Koga H., Tonomura H., Nogi M., Suganuma K., Nishina Y. (2016). Fast, scalable, and eco-friendly fabrication of an energy storage paper electrode. Green Chem..

[102] Balint R., Cassidy N.J., Cartmell S.H. (2014). Conductive polymers: Towards a smart biomaterial for tissue engineering. Acta Biomater..

[103] Herrasti P., Dıaz L., Ocón P., Ibáñez A., Fatas E. (2004). Electrochemical and mechanical properties of polypyrrole coatings on steel. Electrochim. Acta.

[104] Marcilla R., Ochoteco E., Pozo-Gonzalo C., Grande H., Pomposo J.A., Mecerreyes D. (2005). New organic dispersions of conducting polymers using polymeric ionic liquids as stabilizers. Macromol. Rapid Commun..

[105] Andrews R., Jacques D., Qian D., Rantell T. (2002). Multiwall carbon nanotubes: Synthesis and application. Acc. Chem. Res..

[106] Yang J., Rannou P., Planès J., Proń A., Nechtschein M. (1998). Preparation of low density polyethylene-based polyaniline conducting polymer composites with low percolation threshold via extrusion. Synth. Metals.

[107] Koga H., Saito T., Kitaoka T., Nogi M., Suganuma K., Isogai A. (2013). Transparent, Conductive, and Printable Composites Consisting of TEMPO-Oxidized Nanocellulose and Carbon Nanotube. Biomacromolecules.

[108] Tian X.L., Yang C., Si L.L., Si G.L. (2017). Flexible self-assembled membrane electrodes based on eco-friendly bamboo fibers for supercapacitors. J. Mater. Sci.-Mater. Electron..

[109] Zhang L., Zhu P., Zhou F., Zeng W., Su H., Li G., Gao J., Sun R., Wong C.-P. (2015). Flexible asymmetrical solid-state supercapacitors based on laboratory filter paper. ACS Nano.

[110] Liu L., Niu Z., Zhang L., Zhou W., Chen X., Xie S. (2014). Nanostructured graphene composite papers for highly flexible and foldable supercapacitors. Adv. Mater..

[111] Zoski C.G. (2002). Ultramicroelectrodes: Design, fabrication, and characterization. Electroanalysis.

[112] Li B., Zheng M.B., Xue H.G., Pang H. (2016). High performance electrochemical capacitor materials focusing on nickel based materials. Inorg. Chem. Front..

[113] Liu J., Zhang J.G., Yang Z., Lemmon J.P., Imhoff C., Graff G.L., Li L., Hu J., Wang C., Xiao J. (2013). Materials science and materials chemistry for large scale electrochemical energy storage: From transportation to electrical grid. Adv. Funct. Mater..

[114] Zhang S., Pan N. (2015). Supercapacitors performance evaluation. Adv. Energy Mater..

[115] Burke A., Miller M. (2010). Testing of electrochemical capacitors: Capacitance, resistance, energy density, and power capability. Electrochim. Acta.

[116] Linzen D., Buller S., Karden E., De Doncker R.W. (2005). Analysis and evaluation of charge-balancing circuits on performance, reliability, and lifetime of supercapacitor systems. IEEE Trans. Ind. Appl..

[117] Liu Y., Zhou J., Tang J., Tang W. (2015). Three-dimensional, chemically bonded polypyrrole/bacterial cellulose/graphene composites for high-performance supercapacitors. Chem. Mater..

[118] Wang Z., Tammela P., Zhang P., Strømme M., Nyholm L. (2014). High areal and volumetric capacity sustainable all-polymer paper-based supercapacitors. J. Mater. Chem. A.

[119] Thomas B., Raj M.C., Joy J., Moores A., Drisko G.L., Sanchez C.m. (2018). Nanocellulose, a versatile green platform: From biosources to materials and their applications. Chem. Rev..

[120] Gao Y., Jin Z. (2018). Iridescent chiral nematic cellulose nanocrystal/polyvinylpyrrolidone nanocomposite films for distinguishing similar organic solvents. ACS Sustain. Chem. Eng..

[121] Lombardo S., Eyley S., Schütz C., Van Gorp H., Rosenfeldt S., Van den Mooter G., Thielemans W. (2017). Thermodynamic study of the interaction of bovine serum albumin and amino acids with cellulose nanocrystals. Langmuir.

[122] Ruiz-Palomero C., Benítez-Martínez S., Soriano M.L., Valcárcel M. (2017). Fluorescent nanocellulosic hydrogels based on graphene quantum dots for sensing laccase. Anal. Chim. Acta.

[123] Weishaupt R., Siqueira G., Schubert M., Kämpf M.M., Zimmermann T., Maniura-Weber K., Faccio G. (2017). A Protein-Nanocellulose Paper for Sensing Copper Ions at the Nano-to Micromolar Level. Adv. Funct. Mater..

[124] Dubey A., Adhikari N., Mabrouk S., Wu F., Chen K., Yang S., Qiao Q. (2018). A strategic review on processing routes towards highly efficient perovskite solar cells. J. Mater. Chem. A.

[125] Yang Q., Yang J., Shi Z., Xiang S., Xiong C. (2018). Recent progress of nanocellulose-based electroconductive materials and their applications as electronic devices. J. For. Eng..

[126] Gao L., Chao L., Hou M., Liang J., Chen Y., Yu H.-D., Huang W. (2019). Flexible, transparent nanocellulose paper-based perovskite solar cells. Npj Flex. Electron..

[127] Mazloum-Ardakani M., Arazi R., Mirjalili B.B.F., Azad S. (2019). Synthesis and application of Fe3O4@ nanocellulose/TiCl as a nanofiller for high performance of quasisolid-based dye-sensitized solar cells. Int. J. Energy Res..

[128] Chen W., Yu H., Lee S.-Y., Wei T., Li J., Fan Z. (2018). Nanocellulose: A promising nanomaterial for advanced electrochemical energy storage. Chem. Soc. Rev..

[129] Kim J.-H., Lee D., Lee Y.-H., Chen W., Lee S.-Y. (2019). Nanocellulose for Energy Storage Systems: Beyond the Limits of Synthetic Materials. Adv. Mater..

[130] Zhang Y., Zhang L., Cui K., Ge S., Cheng X., Yan M., Yu J., Liu H. (2018). Flexible electronics based on micro/nanostructured paper. Adv. Mater..

[131] Wang Z., Pan R., Sun R., Edstrom K., Strømme M., Nyholm L. (2018). Nanocellulose structured paper-based lithium metal batteries. ACS Appl. Energy Mater..

[132] Pan R., Xu X., Sun R., Wang Z., Lindh J., Edström K., Strømme M., Nyholm L. (2018). Nanocellulose modified polyethylene separators for lithium metal batteries. Small.

[133] Jiao F., Edberg J., Zhao D., Puzinas S., Khan Z.U., Mäkie P., Naderi A., Lindström T., Odén M., Engquist I. (2018). Nanofibrillated Cellulose-Based Electrolyte and Electrode for Paper-Based Supercapacitors. Adv. Sustain. Syst..

[134] Xu H., Hu X., Yang H., Sun Y., Hu C., Huang Y. (2015). Flexible Asymmetric Micro-Supercapacitors Based on Bi2O3 and MnO2 Nanoflowers: Larger Areal Mass Promises Higher Energy Density. Adv. Energy Mater..

[135] Wang Z., Tammela P., Zhang P., Huo J., Ericson F., Strømme M., Nyholm L. (2014). Freestanding nanocellulose-composite fibre reinforced 3D polypyrrole electrodes for energy storage applications. Nanoscale.

